# Tropics and frontier in the writing of Sérgio Buarque de Holanda

**DOI:** 10.1590/S0104-59702023000100008en

**Published:** 2023-04-03

**Authors:** Luiz Feldman

**Affiliations:** i Diplomat, Embassy of Brazil in Mexico. Mexico City – Mexico. otfcgs@gmail.com

**Keywords:** Sérgio Buarque de Holanda (1902-1982), Gilberto Freyre (1900-1987), Jaime Cortesão (1884-1960), history, space

## Abstract

From the mid-1930s, with *Raízes do Brasil* , to the mid-1960s, with *O extremo Oeste* , Sérgio Buarque de Holanda undergoes a significant change in his understanding of Brazilian space. Initially, in a close dialogue with Gilberto Freyre, the author conceives the country drawing on the notion of the tropics, a fluid space where Portugal could be recreated through the bond with the ocean. In *Monções* and *Caminhos e fronteiras* , the historian develops a deliberately opposed vision, conceiving the country from the notion of frontier, a rough space where a foreigner’s adaptability reaches its limit. In this phase, Jaime Cortesão and his thesis of Brazil-island became an invariable target of criticism.

As a war prisoner in Lübeck, several years after having lived in São Paulo, the French historian Fernand Braudel recalled his Brazilian readings to write a brilliant critical analysis of Gilberto Freyre’s work. The text, sent to Lucien Febvre in April 1942 and published in Paris the following year, singles out the writer from Pernambuco as the main name in the essayistic tradition from Euclides da Cunha to Paulo Prado and to Sérgio Buarque de Holanda. He recognizes in Gilberto Freyre’s “geography of the past” the merit of revealing how an intense oceanic experience had welded Brazil to the “maritime immensity;” but criticizes it for not addressing how, inversely, the country had turned to its “continental depth” when losing the impetus of the Atlantic life ( Braudel, 1943 , p.20). The author of *Casa-grande & senzala* (The Masters and the Slaves) dedicated to the sedentary of the coast “all the treasures of his erudition and interpretations. … Thus, Gilberto Freyre’s work, for its massiveness, is basically an appeal in favor of the rooted, the stable ones” ( Braudel, 1943, p , p.8). Braudel noted the need of a “horizontal broadening” (p.19) of perspective, incorporating the nomads, the floating population, the explorers and so many others to whom the country owed its extension and unity.

Having been away from Brazil for five years, possibly Braudel was not aware that his suggestion of telling the history of the explorers had been pursued, not by Gilberto Freyre, by then rather complacent about his own oeuvre, but by Sérgio Buarque de Holanda, unsatisfied with his own writings. In one of his last interviews, published forty years after Braudel’s critical review, Sérgio Buarque points out that the book *Monções* (Monsoons), finished in October 1944 and published in the following year, shaped an idea that was being developed for some time in shorter essays: “To write … a sort of *Casa-grande & senzala* in reverse. Freyre’s book makes Brazil seem static; dominated by sugar; looking at the Atlantic; still. I wanted something more dynamic, pointing to the mines, the inland. Brazil in movement” ( Holanda, 2009, p , p.205). This recollection adequately reflects the program that the historian from São Paulo actually started to outline in a short paper, back in 1939, when army officer Fernand Braudel still patrolled the Maginot Line. By seeking to produce a *Casa-grande & senzala* “in reverse” – and to use the precise expression of the former member of the French mission to the University of São Paulo– Buarque tilted the “geography of the past” from the oceanic welding to the continental immenseness.

In this paper, I discuss Sérgio Buarque’s notion of space and show that his work, between the mid-1930s and mid-1960s, described a movement from the “tropics” to the “frontier.” When *Raízes do Brasil* (Roots of Brazil) was launched in 1936, it was broadly under the influence of *Casa-grande & senzala* , published three years earlier. It is following extensively – though not fully – a formulation by Gilberto Freyre that Sérgio Buarque presents, in *Raízes do Brasil* , both the central problem and the exceptional solution that shaped the country’s historical formation: the adversity of the tropical environment for the European civilization and the adaptability, unique among the peoples of that continent, with which the Portuguese conquered the tropics. Tropical is the “fluid” space where Portugal and its overseas installations could be recreated through the close bond with the ocean. In this coastal environment, no communion is had or pursued with the land. It is, by definition, the exile.

Inverting *Casa-grande & senzala* imposed breaking the mold in which *Raízes do Brasil* had been shaped. The aforementioned article of 1939 initiated the “horizontal broadening,” which *Monções* developed and *Caminhos e fronteiras* (Routes and frontiers) and *O* e *xtremo Oeste* (The Far West) concluded. In these books, the importance of the continental formation of Brazil is deliberately affirmed, against the history or sociology of the maritime shore that had its bastion in Gilberto Freyre. Now in dialogue with authors oriented toward the land – such as Frederick Jackson Turner, Euclides da Cunha, Capistrano de Abreu and Sérgio Milliet – Sérgio Buarque changes the sign of the notion of adaptability from the second edition of *Raízes do Brasil* onward. Instead of producing similarity, meaning recreating the Portuguese ambiance in the tropics, adaptability leads to difference; in other words, it creates a specifically Brazilian society as it advances into the backlands, beyond the Tordesillas Line. ^1^ The frontier is the “rugged” space where adaptability reaches its limit. Jaime Cortesão, a Portuguese exile, becomes a privileged interlocutor at this moment, when Sérgio Buarque seeks to explain the conquest of the “heart” of the South American continent as a result of a spontaneous expansion of the explorers from São Paulo, the *paulistas* , who advanced up to where their organic growth took them, rather than where the Portuguese imperial desire would have inspired them to go. Having emerged in the debate with Jaime Cortesão, this distinction between authentic expansion and geopolitical artificiality became a fundamental aspect of the discussion about frontiers, present in *Caminhos e fronteiras* and extended in part of *História geral da civilização brasileira* (General History of Brazilian Civilization) and in the book *O extremo Oeste* .

The approach proposed in this paper addresses two relatively well-established viewpoints in historiography. First, it contradicts the tendency to attribute to Sérgio Buarque a certain autonomy in relation to Brazilian social thought, especially to Gilberto Freyre’s work (cf. Novais, 2005 ). In the reading presented here, the *editio princeps* of *Raízes do Brasil* bears no marks of anti-Lusitanian emphasis (cf. Candido, 2006 ), which the author only developed from 1940 onward, or of revolt against climate determinism (cf. Dias, 1985 ), which was also a later option, or, finally, of criticism to Freyre’s traditionalism (cf. Bastos, 2016 ), an idea presented in a rereading by Sérgio Buarque of a part of his first book aimed at Alceu Amoroso Lima (1932) as though his original target had been the writer from Pernambuco (cf. Holanda, 1967 , p.7). I shall stress an approach that points to the substantive convergence of the argument of the first *Raízes do Brasil* with that of *Casa-grande & senzala* . ^2^ This point of view, with its antiquity ( Ricardo, 1959) and controversies ( Rocha, 2005) , has been revisited in recent years (Venancio, Wegner, 2018; Sanches, 2021) . In this perspective, the “profoundly static vision, on the verge of stagnation, of Brazilian history” that Ettore Finazzi-Agrò (2005, p , p.147-148) identified in *Raízes do Brasil* – redirecting to Sérgio Buarque, in a sense, Braudel’s criticism to Freyre – seems closely related to the input of *Casa-grande & senzala* . This is the framework within which I seek to outline the notion of the tropics in the work of the author from São Paulo.

Second, the framing presented here contrasts with the evaluations that the spatial element only emerged in Sérgio Buarque’s works that followed *Raízes do Brasil* (cf. Guimarães, 2008 ) and that, even then, the components of his notion of frontier would be mainly metaphorical (cf. Decca, 2006 ) or anthropological (cf. Seabra, 2010 ). As to the supposedly late spatial interest, I agree with Laura de Mello Souza (2014 , p.28) that Sérgio Buarque “was among the historians who sought to move away from the coast and the ‘civilization’ created there to be concerned with the penetration into the territory,” though not without stressing that moving away from the coast was equivalent to abandoning the perspective of *Raízes do Brazil* . As to the meanings of frontier, it is important to register that the dimension of Sérgio Buarque’s writings highlighted here is specifically spatial; anthropological considerations are certainly present, but as part of an effort to explain the formative process of Brazilian territory. This process occurred took place through expansion and hybridization but also by limitations to movement and cultural interchange. In this regard, it is important to point out the divergence between Sérgio Buarque and Jaime Cortesão – which has been played down through a Solomonic integration of each one’s perspective as distinct viewpoints on the same issue (cf. Novais, 2012 ) –, seems to refer to a more fundamental difference in the manner of conceiving the frontier and, even, the country’s space. My intention in exploring this difference is to recover from neglect the dialogue between these two historians, even as I recognize honorable exceptions in both sides of the Atlantic (Oliveira, F., 2010; Oliveira, T., 2013; Martins, 2017 ) and reaffirm the importance of studying the imbrication of history and geography in the Brazilian social thinking (see Lima, 1999 ; Maia, 2008 ).

The four next sections address Sérgio Buarque’s notion of the tropics, the horizontal broadening of his geography of the past, the logic of territorial formation he sought to reject and, finally, his notion of the frontier.

## The conquest of the tropics

Gilberto Freyre’s egg of Columbus consisted in a simple operation, as Evaldo Cabral de Mello (2002) explains: to transform miscegenation from loss into profit. This ingenious change of perspective on a sociological problem that had consumed Brazilian intelligentsia for decades was followed, I stress, by another important operation in *Casa-grande & senzala* , this turn in the field of geography or meteorology. The issue was not refuting the old climate determinism that stipulated the degeneration of man in the tropical or “torrid zone,” a thesis of immemorial origin and still present in the anathema of Euclides da Cunha (1907, p , p.168) on the “tropical belt that condemns us.” *Casa-grande & senzala* actually intended to open up a consecrating exception: contrary to the peoples of northern Europe, whose degeneration in this zone was well verified, the Portuguese, with their unparalleled adaptability, arose to the condition of a people singularly able and even vocationed to overcome the adversities of this part of the globe and successfully colonize it. This is Gilberto Freyre’s other horizon: the rehabilitation of the tropics by and for those capable of colonizing it, saving at least the lands of the Portuguese crown from that harsh old “moral geography,” that defamed the torrid zone (Freyre, 1940, p.59).

“The Portuguese triumphed where the other Europeans failed,” boasted the writer from Pernambuco: “The first modern society constituted in the tropics with national characteristics and qualities of permanence is of Portuguese formation” ( Freyre, 1933 , p.16). The explanation was in the “fortunate predispositions,” racial, ecological and cultural, engendered by the situation of an “undefined people between Europe and Africa” (p.2, 18). In Portugal, a centuries-old miscegenation of Nordic and Arab peoples, the softening climate, more African than European, and the intense cultural hybridism, had formed a people that distinguished themselves by miscibility, acclimatization, and mobility, attributes condensed in the notion of adaptability. All this made the tropics “natural and congenial zones of expansion” of Portugal ( Freyre, 1953 , p.180). Brazil had been a special case, providing “definite evidence of that aptitude” ( Freyre, 1933 , p.1). Differently from the entrepôts created in India and Africa with commercial purposes, in Brazil Lusitanian imperialism unexpectedly transmuted into “agrarian and sedentary activity in the tropics” (p.xviii). From this moment onward, the Portuguese “became Luso-Brazilian: the founder of a new economic and social order” (p.xviii).

The opening page of *Raízes do Brasil* reveals to the reader the same horizon as that of *Casa-grande & senzala* . The author underlines that: “The truly fundamental fact that we constitute the only successful effort, at a large scale, of transplantation of the European culture to a zone of tropical and subtropical climate” ( Holanda, 1936, p , p.3). ^3^ While we are clearly in the presence of Sérgio Buarque’s “economic style”, and no longer in Gilberto Freyre’s “effuse prose” ( Monteiro, 2000) , there is also less need to insist and polemicize. Whoever wrote about the tropical exceptionalism of the Portuguese in 1936 could do it with greater sobriety than in 1933, when that geographical element had to be rehabilitated against still fresh judgements, like that by Euclides da Cunha, about the “incapacity of mestizos to progress … in a physical environment as tropical Brazil” ( Freyre, 1944, p , p.61). If Freyre had to sustain this exceptionalism for the first time – seemingly building on an intuition of Manuel de Oliveira Lima (1922, p , p.34) –, Sérgio Buarque (1936, p.3) could treat that unique aptitude – “we live an experience with no simile” – as a fact of life.

The “echo” of *Casa-grande* *& senzala* , aptly captured in the first paragraph of *Raízes do Brasil* ( Rocha, 2005, p , p.111), extended to the entire discussion of the colony in the book, i.e., until the fifth chapter. This whole segment is pervaded by the notion of the tropics and by Gilberto Freyre’s approach, which I will now circumscribe. Chapter two, which in the Brazilian editions appear with the title “Trabalho & Aventura” (Work and Adventure), is especially relevant, “starting with the use of & to connect the two opposites” ( Pesavento, 2005, p , p.56), so much to the taste of the author of *Casa-grande & senzala* ; then, by the fact that Sérgio Buarque followed very closely the explanatory scheme of that essay to clarify how a culture brought from afar had been maintained in an “environment often unfavorable and hostile” ( Holanda, 1936, p , p.3). From the outset, it is a narrative of exceptionalism:

Pioneers in conquering the tropics for the cause of civilization, the Portuguese saw this achievement as their greatest historical mission. And despite all the accusations that can be made against that accomplishment, the Portuguese were not only effective but also natural bearers of that mission. No other Old World people were so well prepared for venturing into regular and intense exploration of lands near the equator, where, in the 1500s, people were believed to degenerate quickly ( Holanda, 1936 , p.19).

The exceptionalism is explained from the spirit of adventure, in some measure related to Portugal being situated in an “indeterminate region between Europe and Africa” ( Holanda, 1936 , p.4), and the adventure is soon made equivalent to an “extraordinary social adaptability” (p.27). This spirit of adaptation and adventure was the “preeminently harmonizing element” (p.24) of the conjunction of races and cultures in the tropical climate (p.24), characterizing the Portuguese by their attributes of acclimatization and miscibility. The capability to accept “what was suggested by the environment” motivated them to “boldly confront Nature’s harshness and resistance” (p.26, 24). The capacity to enter “into intimate and frequent contact with the colored population,” i.e., with the indigenous and the black peoples, explains how the Portuguese “became Americanized or Africanized to a necessary extent” (p.38). Here is why the Portuguese were “unmatched:” “Perhaps more easily than ever before in history, they succeeded in re-creating in Brazil their own environment” (p.25). By recreating the original environment in the tropics, adaptability engendered similarity: “Neither contact nor mixture with native … races has made us as different from our grandfathers from overseas as, at times, we would like to be” (p.15).

This re-creation occurred not by means of an intransigent extension of the homeland, in the Spanish manner, but through what Gilberto Freyre (1940 , p.23-24) later called, perhaps (now him) with *Raízes do Brasil* in mind, a “splendid adventure of dissolution:” “Always self-dissolving into other peoples, to the point of seemingly losing themselves in strange bloods and cultures. But always communicating to them … their essential motivations of life … Adventure of dissolution followed by the taste of routine.” This was Sérgio Buarque’s conclusion about the adventurer and adaptable colonizers: “Their weakness was their strength” ( Holanda, 1936 , p.37). Here we ought to stress the meaning of this sentence in the context of the previous reference to the “equator.” Having mentioned in the beginning of chapter two the sixteenth-century prejudice about the Torrid Zone and illustrating it with a passage of the French cosmographer André Thevet suggesting man’s loss of robustness in the tropics ( Lestringant, 1997 , p.335 note), Sérgio Buarque ended the chapter quoting an old historian of the Dutch colony in Brazil: “Seventeenth-century Europe believed that ‘below the equator there was no sin’ ... Barlaeus, who mentions this saying, comments: ‘As if the line that divides the world into two hemispheres also separated virtue from vice’” (cited in Holanda, 1936 , p.36 note). These passages placed Sérgio Buarque’s Brazil in a space “beyond-the-line”, in the sense discussed by Carl Schmitt (2003) . The German author referred to the replacement of the line by which the Iberian reigns intended to divide the world between them in Tordesillas by the “line of truce” that emerged with the ascension of Protestant powers. This second line separated Europe, committed to regulating and limiting war, from an “overseas” where lands could only be distributed – the Pope’s arbitral authority no longer holding – according to the law of the strongest. Thevet and Barlaeus brought up the paradox that weakness and vice were Portuguese advantages in an overseas abandoned to the state of nature.

The epigraph of chapter two, “Trabalho & aventura,” a passage by Sallust about the first settlers of Rome, suggests the “fluidity” of the conquest of the tropics and, at the same time, a certain spatial containment in the process: “It is remarkable to note how, after having been gathered within one surrounding wall, they were so easily bound” (cited in Holanda, 1936 , p.17). In fact, the colonization is always “above all coastal and tropical” ( Holanda, 1936, p , p.68). On the coastal dome, the Portuguese became sedentary and were not very concerned about “populating and becoming acquainted with lands beyond the coastal region” (p.79). This only reinforces the mercantile character of their imperialism. The very rural activity is less agriculture than farming, seeking benefit from the land rather than having zeal for it.

Even during the nineteenth century, according to Sérgio Buarque, there was no great variation in this circumstance. Let us take the theme of cordiality. The cordial man had been born in the manor house, the son of the coastal patriarchate (see note 1). The drama of his absorption into the intimacy of the family life, which made him incapable of the impersonalism of public life, develops from the advance of urbanization, especially after the abolition of slavery. However, this palpitating transition in Brazilian life, this change “from one pole to another” ( Holanda, 1936 , p.43), from rural to urban, was a displacement within the spatial circle of the tropics, with both farm and city life being coastal. The references to the world formed by plantations in the backlands or to the new moment in history with the expansion by the *bandeirantes* movement, which “did not have its roots across the ocean” (p.72), were marginal and did not broaden this narrative.

Later, when Sérgio Buarque sought to overcome the coastal geography of *Raízes do Brasil* , he provided some important keys for the understanding of his reasoning about uprootedness. By writing in the preface of *Monções* what his proposal was not, the author claimed he was uninterested in the study of a society that would “only be conservative of a traditional legacy born in a strange climate” ( Holanda, 1945 , p.7). In 1939, “Caminhos e fronteiras” (Routes and frontiers) had formulated it thus:

The other populated sites are not more than dispersed spots afar from the sea, badly planted on the land and almost independent from it. Their locations are better accommodated for the docking of ships than for a good access to the heartland, as if their existence were exclusively directed to the other side of the Atlantic. Here, the Portuguese sought to provoke an environment adapted to their national traditions and their African and Asian experience. The process evolves due to the import of sugar cane. The cultivation of cane generated the sugar mill. And the mill called the negro. This is the frame within which the Portuguese colonization appears in its various nuances in a large extension of the coast. Later, it could serve as a pattern to almost all durable installations of Europeans in the tropics ( Holanda, 1939 , p.14).

Instead of adapting to the land, suffering the discomfort of delving into continental depths, the Portuguese provoked a setting at the seashore that, being frequented by naval squadrons and mercantile fleets that connected it to other entrepôts of a global maritime empire, could adapt to metropolitan traditions , to the comforts and luxury arriving from Portugal and from the East, as well as to the African work force necessary for a mode of production that was brought “ready-made” from the Atlantic islands ( Holanda, 1936 , p.26; see Pesavento, 2005 , p.57). This was the uprootedness mentioned on the initial page of *Raízes do Brasil* : “We have brought our forms of association, our institutions, and our ideas from distant countries, and though we take pride in maintaining all of them in an often unfavorable and hostile environment, we remain uprooted in our own land” ( Holanda, 1936 , p.3). There is no paradox at all (cf. Rocha, 2005 ; cf. Rouanet, 2006 ), but rather coherence, in affirming the successful transplantation of the European culture to the tropics and in noting the condition of uprootedness, a condition initiated, to be precise, in the very beginning of the colonization (cf. Feldman, 2016 ). Neither is there, in this uprootedness, a sense of condemnation. Exile is not uprootedness, but rather the land. As could be read in *Os sertões* (The Backlands): “The land is the unbearable exile” ( Cunha, 1903 , p.143).

The metaphor of rootedness is commonly understood as identity with the land, but in *Raízes do Brasil* it designated the sedentary life on the coast, a life dependent on the ocean. The appropriate metaphor of the roots of Brazil would be that of the manor house and the slaves-quarter, and hence the order they established. However, if *Casa-grande & senzala* admitted some attachment of the colonizer to the land, *Raízes do Brasil* insisted on his exclusive bond with the sea. The colonizer saw no difficulty with a way of life “almost independent from the land” and with “the roots on the other side of the ocean.” The tropics meant “similarity” ( Bastos, 1998) , the globalized similarity between the populated sites in America, Africa and Asia interconnected by the maritime empire and harmonized by the same spirit of adaptation. To take root and to be uprooted were the same. The ideal of self-identity could only be provided through contact with the land, but this contact could only emerged in movement. One had to abandon *Raízes do Brasil* to reach the true roots.

## A line of trading posts and the wilderness

Immediately following the launch of *Raízes do Brasil* a number of meaningful indications can be found about the path taken by Sérgio Buarque. In 1937, Freyre launched *Nordeste* (Northeast), in which he maintained that the civilization of sugar had been “more creative” than that of the mines and of the frontier, having originated and protected the “overflow of effort” represented by the *bandeirismo* ( Freyre, 1937 , p.220, 30). One of the early responses to this book was a review by Sérgio Milliet (1937) , to whom the sugar civilization was associated to luxury and permanence, in contrast to the mobility and poverty of the civilization of São Paulo. He criticizes Freyre for treating the “Brazilians in general” in relation to the characteristics of the people from the Northeast. The reviewer points out that the thesis limits tropical space to the Northeast: “Either [Freyre] removes from the concept of national all territory situated to the South of the tropical line or he denies its inhabitants the right to use the Brazilian nationality” ( Milliet, 1937 , p.45; see also Milliet, 18 nov. 1936, p.4). In 1938, Milliet publishes *Roteiro do café* (Coffee itinerary), with new criticism of Freyre’s generalization, and holds that Brazil had “regional social formations” ( Milliet, 1941 , p.155). In this book, reissued in 1939 and 1941, he endorses the reading of *Raízes do Brasil* (already reviewed by him) about the Portuguese as colonizers averse “to the hard and slow work on the land” (p.126-127).

In March 1939, when publishing the article “Caminhos e fronteiras,” Sérgio Buarque probably knew the criticism of Freyre made by his friend Milliet. In this article, Sérgio Buarque follows Milliet’s line, opposing fixity and opulence in the Northeast to movement and scarcity in São Paulo and circumscribing the sociological range of the tropics to “the society constituted on the coast, especially on the Northeastern coast” ( Holanda, 1939 , p.14). His approach was less on regional diversity and more on the contrast between the coast and the heartland, two “landscapes equally significant” (p.14). From his argument it is inferable that Freyre’s thesis did not necessarily apply to the entire country. It is noteworthy that Milliet (1941 , p.126) was not cited in the article, perhaps not to endorse *Roteiro do café* , in which the author explained the Portuguese lack of interest in agriculture due to “racial motive, as suggested by Sérgio Buarque de Holanda.”

Sérgio Buarque thoroughly pursued the canonical formulation of his new perspective. The image of “Caminhos e fronteiras” had already been quite suggestive and it was unlikely that the contrast with the pair “manor house and slaves-quarter” (literally, in Portuguese: *casa-grande and senzala* ) would be casual in such a conscientious writer. In *Monções* , of 1945, he finds the proper words to enunciate the change – even more than enlargement – of horizon in his work, which he will repeat word by word in the openings of *Caminhos e fronteiras* , of 1957, and (with some alteration) of *O extremo Oeste* , an unfinished work written from 1965 onward (see Holanda, 2014a, p.286 note). As pointed by Robert Wegner (2016) , the key part of this new geography reiterates the terms of the 1939 article about the coast, mentioned above, and affirms:

Once the scabrousness of Serra do Mar is surpassed, especially in the region of Piratininga, the colonial landscape gains a different coloring. Here there is not the external cohesion, the apparent balance, though often fictitious, of the nuclei formed on the Northeastern shore, on the lands of *massapê* soil, where the agrarian wealth is expressed by the solid manor house. The society constituted on the plateau of Martim Afonso’s captaincy remains for a long time in a situation of instability and immaturity, which gives space for greater intercourse of foreigners with the native population. Its vocation would be the route, which invites to movement; not the large property, which creates sedentary individuals ( Holanda, 1945 , p.12-14, 1957, p.I-II; cf. Holanda, 2014b, p.33-34).

The order of the manor house is “fictitious.” There was no longer any room for diplomacy in the manner of 1939, when the “landscapes” of the coast and the heartland where placed as equal. *Monções* opened the perspective of opposition between an artificial and decadent society at the coast and an opulent and vibrant world in the heartland, inhabited by a mestizo race different from the seashore Luso-Brazilians. There was no mention to Freyre, but *Monções* was aimed at his vision – and not only *Monções* . Mobility, absent from *Raízes do Brasil* , became a critically important factor when projected on (the history of) the continent. São Paulo is presented, in this context, not only as the “center of a detailed road network, expanding in all directions of the heartland and the coast” ( Holanda, 1939 , p.14), but also as the result of an exceptional spatial move to overcome the coastal strip.

Inverting *Casa-grande & senzala* imposed weakening its bases in all fronts. After Freyre’s criticism, in June 1940, of the “illustrious essayists and sociologists Sérgio Buarque de Holanda and ... Sérgio Milliet” ( Freyre, 1940, p , p.82) for denying that the Portuguese had any agricultural aptitude, Sérgio Buarque did not hesitate to openly criticize – probably for the first time – his colleague’s theses. In articles of October and November 1940, he explains that Milliet’s reading was “imprecise” in the racial issue but otherwise recognizes agreement with him on the issue of the colonizer’s agrarian inaptitude; he then relativizes Portuguese exceptionality, pointing to similar qualities of the French; and even insinuates limitations in Freyre’s essays, “badly composed” (Holanda, 2011a, p.191, 193). Four years later, in *Cobra de vidro* (Glass Snake), he reverses the formula about the value of the Portuguese adaptability in national formation: “Their strength was their weakness” ( Holanda, 1944, p , p.76). In an article of 1946, he observes that the Portuguese, though having discovered the falseness of the “old superstitions about the Torrid Zone being incompatible with the European civilization” (Holanda, 2011a, p.287), did not take advantage of their vanguard in Brazil. In 1950, Sérgio Buarque shot the poisoned arrow that, in certain historians, “imagination is devouring and consumes all documentation” (Holanda, 2011b, p.25). In *Visão do paraíso* (Vision of Paradise) *,* he notes that the fervent rehabilitation of the tropics was a compensatory exaggeration, from the character Brandônio of *Diálogos das grandezas do Brasil* (Dialogues of Brazil’s Greatness) to the friar Vicente do Salvadr in *História do Brasil* (History of Brazil) – and, as could be read between the lines, to the “Luso-Tropicalism” of Freyre, then at its heyday ( Holanda, 1958 ; cf. Freyre, 1953) .

Unsurprisingly, the revisions of *Raízes do Brasil* in 1948 and 1956 crucially suppress parts of the book that were underpinned by several aspects of the thesis of *Casa-grande & senzala* – climatic, racial, landscape, linguistic and liturgical (cf. Holanda, 1936, p , p.3, 28n, 62, 104n, 105). From the perspective of the ideal of identity with the land, the condition of uprootedness, previously a relatively uncontroversial description of the primacy of coastal society, becomes the manifestation of a fundamental perplexity of *Raízes do Brasil* with something that *Os sertões* had identified decades before as the “anomaly of displacing to a new land the moral environment of an old society” ( Cunha, 1903, p , p.81). And it is meaningful that directly after this passage, Euclides da Cunha praised the importance of Serra do Mar, the mountain range along the ocean, in national history: “Once the mountain was transposed – bowed as the stone belt of a continent – it was an ethnical isolation and a historical isolation. It nullified the irrepressible attachment to the coast” (p.82). Facing and surmounting the arduous way up the mountain was an “exception” (Holanda, 2011b, p.240) in the history of coastal Brazil; but it meant leaving behind the exile and transforming the sedentary way of life of the seashore into one opposed to the authentic values generated by the intimate contact with the land. Furthermore, it meant the possibility of a new ethnical profile, as will soon become clear.

The first *Raízes do Brasil* acknowledged “all that can and should be alleged” against the work of the Portuguese in the tropics, but it did describe with strong emphasis their efficiency (“effective” conveyors of the colonizing mission). Revised in 1948, the book presents at the end of the fourth chapter a long digression about the Lusitanian “landscape of decline” during overseas expansion and even before ( Holanda, 1948 , p.164). Against the epic colors of Camões in *Os lusíadas* (The Lusiads), for example, the author recommended Diogo do Couto’s reflection in *Soldado prático* (The Practical Soldier), about why “great empires are brought to a fall” (cited in Holanda, 1948 , p.163). This new panorama of chapter four works as a counterpoint to the lively scenery composed on a long note then added at the end of the chapter. The *paulistas* revealed a “vast and opulent world” in the heartland , in contrast to the “little more than a line of fortresses and trading posts ten thousand miles long” (Tawney cited in Holanda, 1948 , p.192), a definition that accentuated the oceanic and commercial characteristics of the Portuguese colonial empire. Together with the previous argument that there had never been agriculture, this reinforced the statement, already occurring in 1936, that the Portuguese presence in Brazil “had a more accentuated character of setting up trading posts than of colonization” ( Holanda, 1948 , p.152; see Holanda, 1936 , p.80). The relatively marginal difference from *Casa-grande & senzala* in 1936 became a considerable divergence in 1948. The Portuguese settlement in the tropics was not a colony based on the order of the manor houses that cultivated the land, but instead a line of trading posts that devastated it.

With this opposition between the coastal line and the world of the heartland, which brought to the revised edition of *Raízes do Brasil* the continental deepth that was missing in the first edition, Sérgio Buarque was transferring the protagonism of Brazilian history to inland society, as had been previously done by Capistrano de Abreu ( Schwartz, 1997 ). As he distanced himself from Freyre, a thinker of the maritime expansion, Sérgio Buarque developed a creative dialogue with thinkers of the land. This occurs not only with Euclides da Cunha, but also with Capistrano de Abreu, whose image of a “leather era” (Abreu, 1907, p.128) in the backlands inspired the beautiful metaphor of adaptability in *Monções* , repeated in the books that followed:

Developing with greater freedom and abandonment than in other captaincies, the colonizing action is performed here by a process of continuous adaptation to specific conditions of the American environment. For this reason, it is not built at once in inflexible forms. On the contrary, it retrogresses to rude and primitive patterns: a sort of tribute required for better knowledge and the final possession of the land. Only very slowly, though with extraordinary consistency, does the European succeed in implanting in a strange country some forms of life already familiar in the Old World. With the consistency of leather, not of iron or bronze, bending, adjusting, molding to all the ruggedness of the soil ( Holanda, 1945 , p.12-14).

It has been demonstrated that the “frontier thesis” of the North American historian Frederick Jackson Turner inspired this argument about the foreign colonizer who leaves behind his way of life, assimilates indigenous uses and customs, and only slowly introduces foreign ways of life into his new environment, eventually engendering an original culture ( Wegner, 2000 ). Indeed, after getting to know *The frontier in American history* , in 1941, Sérgio Buarque not only started to consider the possibility of a “Brazilian Turner” ( Holanda, 2018 , p.22), but also concludes that Turner’s thesis was even more valid for Brazil than for the United States. The Tupi-Portuguese bilingualism and the adoption of indigenous methods of riverine transport suggested that the European “totally compromised with the indigenous processes” ( Holanda, 1957 , p.202), to a degree that, according to Turner’s criteria (1920), in the United States it would only have been achieved with scandal.

It is noteworthy that the focus on the adaptation of the European way of life to American environment – and no longer on the adaptation of the American environment to European way of life – involves a change of sign in adaptability: in the tropics, it generates similarity; on the frontier, it generates difference. This notion stands out in the 1948 edition of *Raízes do Brasil* : “In their capacity to adapt to all environments – at the cost, at times, of their own racial and cultural characteristics – the Portuguese revealed themselves better colonizers than other peoples” ( Holanda, 1948 , p.192). The rusticity of São Paulo’s society, which lacked the stable means of survival of the Northeast, forced mobility toward the heartland. While domain over the heartland could not have been achieved without the help of the natives, the Portuguese “could not survive with them in a pure state” (p.191). In *Caminhos e fronteiras* , Sérgio Buarque wrote: “These places created a race in many aspects closer to the *bugre* than to the European” ( Holanda, 1957 , p.145; see Holanda, 1949 , p.280). This was the crucial transformation, from Europeans into Americans, as quoted from Georg Friederici: “The whole vast heartland of Brazil was discovered and revealed to Europeans not by Europeans but by Americans instead” (cited in Holanda, 1948 , p.191-192).

The continental deepth, though opulent, soon was revealed as “arduous and hostile” ( Holanda, 1949 , p.183), endangering man with “arrows,” “beasts” and “fevers” ( Holanda, 1945 , p.202). The long dealings with the reality of the heartland, as well as miscegenation, taught the *paulistas* how to obtain their ways of subsistence in harmony with the animal and vegetal environment. This turned them into conquerors with the “mark of the wild calling” inside themselves and, therefore, having “powerful bonds” with the “new land” ( Holanda, 1949 , p.180). Their adaptation was so complete, in the sense of accepting primitive ways of life, that the *paulistas* developed a “sense of community and even kinship with other natural beings” and the “perfect integration in a treacherous and wild world” (p.227). In sum, adaptability led the explorer who delved far beyond the Line of Tordesillas to an absolutely authentic way of life: “The true source of active energies” was not in the moderate customs of the coast, but “in the obscure instincts, the mostly rude inclinations, and the often immoral interests that animated the *bandeirante* unraveller of the heartland” (p.180-181).

On the one hand, artificiality and decay of the coastal strip; on the other hand, organic life and a peculiar ethnic profile in the backlands world. It is based on these presuppositions that Sérgio Buarque will explain the spontaneous expansion of the *paulistas* into the continent.

## The natural frontiers

The influent and prolific author whom Sérgio Buarque privileged as interlocutor about this process was the Portuguese Jaime Cortesão. By the early 1950s, as both held a debate in the press after Sérgio Buarque challenged his thesis of the “myth of the Brazil-island,” Cortesão was the only foreigner ever admitted in the faculty of Rio Branco Institute, the Brazilian diplomatic academy, where he lectured cartography and territorial formation. This is not the occasion to reconstitute this debate (see Oliveira, 2010 ), though I do point out that the initiative seemed to always come from Sérgio Buarque; not only in the controversy in the press in 1952, but also in its fermentation since some time before (see the simultaneous publications in the newspaper *O Estado de S. Paulo* in 1948: Cortesão, 1964a; Holanda, 2011a) and, years later, in the unilateral continuation of the antagonism. By 1958, for example, Sérgio Buarque already intended to prolong his stand on the debate by making it part and even the title of a book, as can be inferred from the list of works in his habilitation thesis ( Holanda, 1958 ), where it appears as “in print” with the title *Tentativas de mitologia: estudos brasileiros* (Attempts of mythology: Brazilian studies), a book published (without this subtitle) 21 years later ( Holanda, 1979 ). The book *Visão do paraíso* made critical mention of the idea of a Brazil-island and, in 1960, the same year as Professor Cortesão’s death, Sérgio Buarque resumed the subject in the chapter “A Colônia do Sacramento e a expansão no extremo Sul,” (The Colony of Sacramento and the expansion in the far South), part of the *História geral da civilização brasileira* under his direction. Some five years later, when writing *O extremo Oeste* , he highlights the thesis of the Brazil-island right at the outset, after quoting once more opening paragraphs of *Monções* and *Caminhos e fronteiras* ; he then takes this thesis as motto for the entire discussion of the second chapter, “A conquista do extremo Oeste” (The conquest of the far West). Thus, after the indefectible criticism of Gilberto Freyre, Jaime Cortesão came as the preferred interlocutor (for the dialogue with Afonso Taunay, see Schneider, Martins, 2019).

Sérgio Buarque and Jaime Cortesão had truly different visions of the causes and effects of the expansion westward of the Line of Tordesillas. The exiled professor explained it as a result of the conscious initiative of pioneers of the heartland from São Paulo and from Belém do Pará, simultaneously to that of cartographers and diplomats of Portugal, all aiming at correcting the amputation imposed by the 1494 treaty on the geographic unity of Portuguese America. According to Cortesão, such unity had been foreboded since the sixteeth-century through the mythical image of Brazil as an island that had, to one side, the waters of the Atlantic Ocean, and, to the other, the basins of the La Plata and Amazon rivers, whose arms met at a large lake in the center of the continent (Cortesão, 1964a, 1964b, 1971). Sérgio Buarque, in turn, explained the expansion of the country’s geographic silhouette as an unforeseen consequence of the establishment of a long line of circulation and occupation of the heartland by expeditioners from São Paulo, the land-bound *bandeirantes* and the river-bound *monçoeiros* , a fact on the ground which Portuguese diplomacy had then cunning enough to convert into bargaining advantage in the Treaty of Madrid. The journeys of those pioneers overland, which went increasingly further, were an escape from the poverty that struck them in Piratininga. They sought the “reservoirs” of native work force for agriculture, especially in the west of Paraná River; later, they sought enrichment from mining in Mato Grosso ( Holanda, 1945 , 1946 , 2014b ).

In 1952, Sérgio Buarque sensed his main target in Cortesão’s thesis: “I cannot find, in any of his writings, the notion of the so-called ‘natural limits,’ but it is evident that in his vision it presides that notion of [geographical] unity” ( Holanda, 1979 , p.72-73). In 1958, he already knew a text of 1934 in which Cortesão, despite being doctrinally against geographical determinism ( Oliveira, 2017 ), had made a qualified use of the expression “natural frontiers” to treat the left bank of the La Plata-Paraná-Paraguay rivers and the western border of the Brazilian plateau ( Cortesão, 1971 , p.101, 195; see Holanda, 1958 , p.172 note). In 1960, in the chapter on Colônia do Sacramento, Sérgio Buarque mentions the “myth of the ‘natural frontiers’” ( Holanda, 2007 , p.369), as if he was going into the essence of the “myth of the Brazil-island.” In other words, Sérgio Buarque aimed at a logic of natural frontiers according to which nature would have provided, especially with rivers and lakes, self-evident landmarks to separate Iberian possessions in America. This reading highlighted two differences between his approach and that of Cortesão, one, historical, and the other one anthropological.

In terms of historical approach, Cortesão seemed to presuppose a Lusitanian predestination to occupy the entire territory that later became Brazil, whereas Sérgio Buarque defended a history open to contingency. Cortesão (1964a, p.99) wrote: “The history of Brazil will remain, for the most part, a chaos of incoherent facts, if we do not admit that the intuition and, soon, knowledge of that unity were matched by a unitary policy by both Portugal and the Luso-Brazilians.” And Sérgio Buarque wrote: “In this way, the multiplicity of very small historical incidents, not seldom divergent between themselves, acquires a broad framing … and, clarified by a light that seems to come from above, they receive … the intelligibility that was lacking” (Holanda, 2014b, p.106). In terms of anthropological approach, Cortesão (1964b) wrote about a type of adaptability of the expeditioners that, ultimately, generated similarity and not difference. He did recognize that the *paulistas* connected “gradually with the land” (p.212) and were even “Americanized. Became differentiated” (p.138). But the main thrust of his argument is to highlight similarity. He would go on to claim that the miscegenation of the Portuguese with the Tupi, facilitated by the “similarity” of cultures, given that both were “typically expansionist[s]” ( Cortesão, 1955 , p.126), resulted in a hybrid that merged “the nomad mobility and restlessness to the discipline and imperial sense of space of the Portuguese” (Cortesão, 1964b, p.202).

These lines of thought led to a difference about the mental horizon of the pioneers. For Cortesão, the colonizer knew as much as anyone from the metropolis the interests of the Portuguese empire, whereas for Sérgio Buarque the settler knew better than anyone in the metropolis what the interests of the Brazilian colony were. Cortesão wrote in 1948: Luso-Tupi *bandeirismo* was “illuminated at spaces, as circulating lamps, by the lightings of a superior faith and moral and political awareness” (Cortesão, 1964b, p.228); Sérgio Buarque wrote in 1954: “In the mid-seventeenth century, the sons and grandchildren of the colonizers had learnt, in the New World, to know their needs, to measure their strength and to employ them according to their interest and convenience, which did not always correspond to those of Portugal” ( Holanda, 2002 , p.89). Therefore, in Cortesão, a mobile and congenial imperial awareness enabled consecutive generations of colonizers deliberately to collaborate with the revision of the Line of Tordesillas – thus providing efficiency to empire in utterly unknown continental depths. A picture quite diverse from that of Sérgio Buarque, in which sentences such as that on the Americans revealing the heartland to the Europeans highlighted an “element of political differentiation related to the land” ( Galli, 2015 , p.114). After quoting once more this sentence by Friederici in the 1952 debate in the press, Sérgio Buarque was accused of “Tupi nationalism” ( Cortesão, 1952 , p.1).

In fact, the debate on the press opposed a “man from the colony” ( Russell-Wood, 2009 ) to a historian of overseas expansion. And perhaps Sérgio Buarque could have used a variant of Sallust’s epigraph against Cortesão: “It is amazing to say how easily they have expanded.” It seems to me that, ultimately, this is the premise of “natural frontiers” that Sérgio Buarque highlights in the oeuvre of the Portuguese historian only to entirely negate them: a “fluid” space, in which the undeniable hindrances of westward expansion would perforce be overcome because history wanted it, and because the expeditioners preserved in their souls the empire’s interests even in the face of utmost hardship. Against the image of a territory with self-evident limits, whose progressive occupation was facilitated by historical necessities and anthropological attenuations, Sérgio Buarque presented a “rugged” space, in which the results of the arduous expansion were uncertain and the unrestricted adhesion to native culture was a matter of life or death.

This conception had two relevant implications. One was to confront visions that separated Brazil geographically and culturally from its neighbors. The image of Brazil as an island “that is very clearly separated from the rest of South America” ( Holanda, 1979 , p.78) did not go well with Sérgio Buarque, who was aware of Brazilians’ “indifference” ( Holanda, 1955 , p.74) regarding Spanish America. This concern set the tone of the editorial project that Sérgio Buarque led in *História geral da civilização brasileira* , in which he employed a series of comparisons with the past of other Latin American countries to dilute the singularities of the formative process of Brazilian politics ( Furtado, 2018 ; see Nicodemo, 2013 ). All of this disagreed with an insular Brazil. Perhaps in Sérgio Buarque’s vision, the thesis of a Brazil-island appeared as an extemporaneous resurgence of what *Visão do paraíso* had defined as an “insular romanticism” inherited from the great navigations, whose distinctive trait, from Camões to More, was the conversion of the “poetic aureole” inspired by the vision of solitary islands in the ocean into a “mystical refulgence” ( Holanda, 1958 , p.304).

Another implication, only apparently contradictory with the first one, was to diverge from a certain trend of opinion in the La Plata basin determined to establish that Portuguese territorial aggrandizement in South America, epitomized in the Treaty of Madrid, resulted from diplomatic astuteness and not from military victories. This was the old idea that the “exceptional sinuosity” of Lisbon statesmen, inherited by those in Rio de Janeiro, which had its counterpart in the scarce “superior warrior virtues” of Luso-Brazilian men-at-arms (Holanda, 2014b, p.109), an idea that echoed even in *Raízes do Brasil* (see the image of the “giant full of superior bonhomie” in Holanda, 1936 , p.143). Baron Rio Branco had already taken a stand against this opinion (see Paranhos, 1902 , p.49 note) and Sérgio Buarque deliberately presents himself as his follower. In its most recent incarnation, he noted, that interpretative trend resorted to the very thesis of the Brazil-island to reinforce the argument that Portuguese territorial expansion had been decided with the pen rather than with the sword. Sérgio Buarque wished to clear up misunderstandings both overseas and at home, criticizing the thesis of the Brazil-island both as a source of Portuguese glory in the territorial conquest of America (Cortesão’s meaning) and as a source of dishonor of the *paulistas* in the same activity (in a certain Hispano-American meaning).

## The heart of the continent

For those with a vocation for movement, overcoming the scabrousness of Serra do Mar was only the first stage in the long course toward the final possession of the immense land. From *Monções* onward, Sérgio Buarque ( Holanda, 1945 , p.184) will study extensively the “Luso-Brazilian expansion into the western heartland,” giving special emphasis to the incorporation of what is today Mato Grosso do Sul and Mato Grosso through the dangerous riverine journeys of *monçoeiros* from São Paulo in search of Cuibá mines and their riches. This choice of subject is not aleatory. The conquest of this region is equivalent, he claims in a chapter of the edited book *Curso de bandeirologia* (Course on the study of *Bandeiras* ), to the “integration in the world of our culture of all the immense territory that constitutes the heart of this South American continent” ( Holanda, 1946 , p.145). The heart metaphor is repeated in *Monções* , *Visão do paraíso* and *O extremo Oeste* .

One might then ask, how and in the face of what hardships did the expeditioners move the frontier beyond the Line of Tordesillas, taking possession over the rugged heart of South America? The great obstacle to *paulista* expansion in the eighteenth century through what was then known as the fields of Guairá and Xerez did not come from resistance by the established powers of Spanish America (protagonists in Cortesão’s history). Rather, according to Sérgio Buarque, it took the form of a lethal native alliance of *paiaguás* and *guaicurus* , only to a certain extent fomented by Spanish colonial authorities. *Caminhos e fronteiras* gives the measure of the resistance that awaited the *paulistas* in the region and the type of war that they battled; *O extremo Oeste* elaborates on the military dimension of the adaptability of the *paulistas* ; and a chapter of *História geral* about the Colônia do Sacramento, where the expansion of São Paulo fails, presents the counterproof of the narrative. A symbol of all this narrative is found in *Monções* , in the canoes that navigated the Brazilian rivers until they reached the center of the South American land.

Let us recapitulate the end of the second section. The “perfect integration” of the *paulistas* in the “treacherous and wild world” involved the learning of also treacherous and fraudulent hunting techniques, in which the expeditioner “intends to almost be on a par with the animals and even the trees in the forest with the purpose of misleading and better destroying the prey” ( Holanda, 1957 , p.79-80). It so happened that these means of subsistence were equally valid in the fight for survival; hence, military tactics were an extension of hunting techniques. Conducting hostilities without being exposed and fighting with animal ferocity were expressions of the “adequate type of war for this country” (p.146). But those had nothing in common with the type of limited war, conducted between regular troops, practiced in Europe in the eighteenth century. As opposed to this latter type of conflict, conceived of as a duel and governed by inflexible codes and the moral parity of combatants, in the landscape of colonial war in *Caminhos e fronteiras* “the battle only occurs between mortal enemies” ( Strauss, 2007 , p.121; see Schmitt, 1963 , p.88). Leo Strauss wrote these words to refer to the idea of conflict in Donoso Cortés, and they are pertinent here. Wishing to stress the degree of enmity that pervaded conflict in the heartland, Sérgio Buarque ( Holanda, 1957 , p.143) affirms: “Our rustic man is, often, far from belonging, as certain urban bourgeoisies, to the ‘debater race’ of Donoso Cortez [ *sic* ].” This quote suggests that, far from the destiny of “servitude” that the great Spanish reactionary reserved to the debater races in his 1852 letter referred to by Sérgio Buarque, the expeditioner would be closer to the “warrior races” on which “empire” would be bestowed ( Cortés, 1855 , p.230).

Soon after citing this letter by Donoso, in which acknowledgement was made of “what is fecund about war” ( Cortés, 1855 , p.230), Sérgio Buarque ( Holanda, 1957 , p.145) extolled the “military genius” with which the fearsome *paulistas* went on “expanding in the continent the world of the Portuguese language.” This expansion and also the conquering of “virgin lands” were held to be “good services” (p.145) by the expeditioners of the heartlands. Their “riotous aggressiveness” even acquired, according to the author, “a positive and, at the end of the day, necessary function” (p.102; cf. Schwarcz, Monteiro, 2023, p.14). This reasoning of *Caminhos e fronteiras* , which was already extant in the long article “Índios e mamelucos na expansão paulista” (Natives and mestizos in the *paulista* expansion), of 1949, strongly contrasts with the panorama of Lusitanian decay described in the 1948 revision of *Raízes do Brasil* . Nothing in the “warrior race” and in the “empire” they went on erecting in the heartland bore any similarity to the inactive Lusitanian nobility that, having unlearnt the art of war and “replaced soldiers with scribes” (Couto cited in Holanda, 1948 , p.164), in the words of *Soldado prático* , had dealt a death blow to the Portuguese maritime empire. In *Visão do paraíso* , Sérgio Buarque went to great lengths in order to affirm that Hugo Grotius’s devastating criticism of the Eastern Lusitanian empire in *De iure praedae* also held for the Western empire. Following the Dutchman, he observed that the Portuguese were more based on “treachery” than on violence, given that, though having the same “malice” as the Spaniards, they were weaker and more cowardly and therefor used a “mask of peace and friendship” to perpetrate the “most terrible” crimes ( Holanda, 1958 , p.354-355; cites Grócio, 2006 , p.260). It is noteworthy that these statements are in the antipodes of the vision of Gilberto Freyre, in his Luso-tropical years, on the efficiency and morality of Portuguese imperialism (see Feldman, 2021 ).

Appropriately enough, in this context Sérgio Buarque had started to imagine the heartland as an oceanic vastness crisscrossed by the *monçoeiros* from São Paulo, rightful heirs of the audacious sailors of Eastern monsoons:

The agents and protagonists of this movement left from an inhabited port ‒ Araritaguaba – to reach, five months later, another port – Cuiabá – having crossed a vast and uninhabited area like the ocean. The Camapoã farm, located halfway, is an island where the navigator seeks refreshment and rest. And, if it so happens that the canoes are assaulted by fierce natives, most likely these assaults are carried out by the ferocious Paiaguá, the pirates of the Taquari and the Paraguay rivers ( Holanda, 1946 , p.143, 1957, p.177).

Once they surpassed the mouth of the Coxim River, “a raging cape of the Cuiabá navigation” ( Holanda, 1945 , p.160), the amphibious chasing by the paiaguás, skillful canoeists, ceased, only to be followed by the “realm of the courageous riders” (p.162), meaning the guaicurus, to whom Sérgio Buarque (Holanda, 2014a, p.339), in a revised version of *Monções* , attributed responsibility for the “insularity” in which the mines of Mato Grosso were maintained during most of the eighteenth century.

Referring to these indigenous peoples as pirates meant deploying the utmost concept of enmity ( Schmitt, 2003 , p.65) to describe the “mortal intolerance” (Holanda, 2014b, p.73) they devoted to the *paulistas* . It also meant characterizing an environment as opposed as could be to that perfidious peace between the manor house and the slave-quarter, rejecting the kind of facile interaction and fraternity that both Gilberto Freyre and Jaime Cortesão inferred from miscegenation. This panorama evokes the observation of *The nomos of the Earth* about the ancestral vision of the sea as “impervious to human law and human order, that it is a realm free for tests of strength. That is the meaning of the delimitation of amity lines” ( Schmitt, 2003 , p.181). The exceptionalism of tropical Portuguese colonization, in which weakness was strength, could no longer relativize the law of the strongest in overseas possessions. Here one was entirely in that “beyond-the-line” – both of the equator and of the Tordesillas – in which only unequivocal adaptation to the treacherous environment and abandonment of European conventions, including those of war, could ensure the survival of the colonizer. The fixity of the coastal manor house was fully opposed by the mobility of the canoes that sailed the continent (see Schmitt, 1962 ). Roots were formed on the terrestrial and riverine routes, on the metaphorical and literal navigation of the heartland, and on the slow and violent inscription of an order – of an empire, according to Donoso Cortés – on the land.

In *O extremo Oeste* , Sérgio Buarque mentions approvingly *The Art of War* , by Machiavelli, whose teachings he found useful for the understanding of the conquest of Mato Grosso. In the book, the Florentine extolled the ancient Romans for their wisdom in “preparing the body for discomfort and the mind to be fearless” ( Maquiavel, 2006 , p.224). This seems to be the true worth of the “grim race” ( Holanda, 1945 , p.145) of the *paulistas* , the one that enabled their organic expansion, always close to the land. The occupation of the old fields of Guairá and Xerez by the *paulistas* could not have been effective “if they did not have working for them the continuous exercise of the rugged paths and the sure knowledge of the resources used by the natives themselves in the face of nature’s hindrances and of their enemies’ maliciousness” (Holanda, 2014b, p.68). On the one hand, the *bandeirantes* knew how to accommodate “better to the roughness of some still indomitable corners” than their “Hispano-Guarani” competitors (p.193, 189), who, not used to walking long distances on foot, could not take advantage of their skills as riders in the swampy regions, thus leaving the space open to the settlement of *paulistas* . On the other hand, the *monçoeiros* eventually consolidated their position against the Paiaguás by improving their adaptability with a more adequate naval strategy to face their enemies’ agility and violence. The *bandeirantes* walking on foot, the *monçoeiros* adopting monoxylon canoes, all convincing themselves of the “insufficiency of civilized armaments” (p.68) and of the imperative adaptation to the natives: this was how the “effective occupation of Central Brazil” was achieved (Holanda, 2014a, p.325).

As I have mentioned before, this discussion aimed at overthrowing the notion of Brazilian military inferiority. In doing so, however, Sérgio Buarque ( Holanda, 2007 , p.370) went to the extreme of wanting to eliminate “the slightest indication” that Portugal had ever been inspired by the idea of natural frontiers. Against Cortesão (2006) , he affirms – mistakenly, according to Luís Ferrand de Almeida (1973 , p.318) and Synesio Sampaio Goes Filho (2021, p.116) – that the Treaty of Madrid had been based exclusively on the “more profane notion” of *uti possidetis* ( Holanda, 2007 , p.372). In practical terms, Sérgio Buarque reduced Portuguese diplomatic activity to fixing on the text of that treaty the fact – which he attributed entirely to the action of the *paulistas* – of the territorial possession of the heartland. For Sérgio Buarque, the principle of *uti possidetis* emerges as the judicial coronation of a history of organic expansion. Whereas according to Cortesão (2006) Alexandre de Gusmão, was the competent diplomat capable of combining *uti possidetis* and natural limits to obtain, on the negotiating table, a better result than the objective conditions on the ground in South American indicated, according to Sérgio Buarque ( Holanda, 2007 ) he was no more than a clever statesman, who duly identified the best form to consummate a fact.

From a 1948 conference onward, Sérgio Buarque counterposed movements imposed by the “most rudimentary needs of a population” to “artful limits dictated by diplomatic conveniences” (Holanda, 2011a, p.505). This is how he analyzed the fate of the Portuguese stronghold installed in 1680 on the left bank of the La Plata River, the Colônia do Sacramento. Although soldiers from São Paulo had been sent to establish it, they were neither “owners of the land’s secrets” nor “accustomed to all its privations” ( Holanda, 2007 , p.387) in that bank of the La Plata. The “La Plata adventure” had not been a “truly spontaneous effort,” but rather an artificial action of Portugal, alien to “the internal evolution of Portuguese America” (p.386-388). The effectiveness of territorial expansion was subject to the old organicist logic, according to which the internal law of an organism, in this case the *paulista* culture, needed an external environment to sustain it. Only in that way could there be spontaneity, i.e., the “capacity to grow drawing on itself and … to adapt to the conditions of life” ( Eugenio, 2010 , p.350). If geopolitical injunctions already looked artificial where there organic expansion took place, this would apply even ore where there were no “powerful bonds” with the land: “That piece of land, conquered not by arms but by international contingencies, could only remain in Portuguese hands through decisions usually taken not in America, but rather in the battlefields and diplomatic treaties of the Old World” ( Holanda, 2007 , p.388). To sum up, the same reasons that explained the conquest of the “heart” of Portuguese America explained the loss of its extremity when the circumstances decided this way.

Here we are at the core of Sérgio Buarque’s notion of frontier, as set forth when he discussed the title of *Caminhos e fronteiras* :

If mention is made of the route, ‘which invites to movement,’ this is precisely in order to point out the characteristic mobility, especially in the early centuries, of populations of the *paulista* plateau …, the fact is that this very mobility is conditioned among them and will, on its turn, condition the situation implied in the idea of ‘frontier.’ Frontier, properly understood, between landscapes, populations, customs, institutions, techniques, even heterogeneous languages in confrontation, either subduing and leaving space for the formation of mixed or symbiotic products, or affirming themselves at least while not overcame by the final victory of the elements that turned out to be the most active, most robust or better equipped ( Holanda, 1957 , p.VI).

It has been said that, in Sérgio Buarque, Brazilian territory was formed less through settlements than through movement ( Vangelista, 2005 ). This is true, but movement had a limit, not a natural one, due to some artificial stipulation, but rather organic, marked by the exact extension of the porosity of Piratininga men’s to the environment. The frontier became tenuous, in the sense of the attenuation of contrasts, while there could be adaptability, and was affirmed where this adaptability ended. Here were the limits of the territorial empire of the grim *paulistas* .

## Final considerations

In *Raízes do Brasil* , the sea generated similarity. This was uprootedness in the tropics, a circumstance logically due to Brazil being one of the footholds, on the Atlantic coast of South America, of a global maritime empire. At that time, “penetrating the root of problems” could be, for Sérgio Buarque, to expose the country’s sedentary formation; but it could not be equivalent to being “radically critical in his manner of analyzing trendy notions and concepts, such as patriarchalism, race, Portuguese tradition etc.” ( Candido, 1980 , n/p). In these pages, I sought to demonstrate that the author’s critical turn against this fluid vision of the world, now seen as a coastal exile that looks the other way from the reality of the heartland, is inseparable from his spatial reorientation toward the continental depth. Starting with the article “Caminhos e fronteiras” and the book *Monções* , then followed by the revision of *Raízes do Brasil* , by “Índios e mamelucos na expansão paulista,” the debate on the Brazil-island, by the book *Caminhos e fronteiras* , by *Visão do paraíso* , and the study on Colônia do Sacramento, up until *O extremo Oeste* , the land generates difference. The focus now falls on the rugged frontier, faced by the expeditioners who move Brazil away from an overseas existence and establish its peculiar profile, including in the geographical sense. Along this trajectory, the emphasis on mobility – and its limits – enables a radical approach of sedentariness. The atmosphere of perfidious intimacy of the tropics, which only supposedly built some order, could now be contrasted with the moral education of robustness (proclaimed by Machiavelli) in the face of mortal enmities (hailed by Donoso Cortés), a hallmark of the frontiers taken greatly beyond European lines and conventions by the sailors of the continent. Sérgio Buarque abandoned the “cordial” society on the seashore to approach the telluric one, emerged in the conquest of the “heart” of the land. Thence came the country’s authentic life, there the treasures of its intelligence should be placed.

## NOTES

1 In approaching Sérgio Buarque’s distinct meanings of adaptability, I benefited from the classical thesis by Edmundo O’Gorman (1977) on the geographical and historical “invention” of America.

2 Richard Morse (1995 , p.53) once noted that “the first two chapters of *Raízes do Brasil* fit into many of the ideas of Gilberto [Freyre], though it is difficult to attribute influence, given the robust manuscript that Sérgio [Buarque] brought from Germany in 1931.” This manuscript, entitled “Teoria da América” (Theory of America), of unknown whereabouts, may have entailed an “attempt of mythology” by Sérgio Buarque, who in a homonymous book of 1979 placed it as a sort of mythical origin of *Raízes do Brasil* , associating the article of 1935, that foretells the essay of the following year, to readings during the German season and keeping it relatively safe from correlations with the Brazilian intellectual environment. About the manuscript “Teoria da América,” he writes: “From it, I draw the essential from a history study commissioned by Claudio Ganns for a luxurious new journal. … The title was *Corpo e alma do Brasil* ” ( Holanda, 1979 , p.30). It happens that, both in “Corpo e alma do Brasil” (Body and Soul of Brazil) and *Raízes do Brasil* , Sérgio Buarque presents the “cordial man,” closely associating the propension to shortening social distances and the need of intimacy emerged in the zone of slavery colonization, referred to in *Casa-grande e senzala* (see Feldman, 2016 , p.153-156). This book was qualified in the article as “the most serious and complete essay that has ever been attempted on the social formation of Brazil” ( Holanda, 2006 , p.402). Almost identical praises were made to *Casa-grande* in *Raízes* (see Holanda, 1936 , p.105). There is, therefore, a reason to treat cautiously the focus on the German inspirations of the author of *Raízes do Brasil* in detriment of his national readings upon returning from Berlin (cf. Lima, 2008 ). For, unless one does not consider cordiality an “essential” part – perhaps “the” essential part – of the article of 1935 and the book of 1936, it is possible to suggest that this synthesis-idea of the Portuguese colonial legacy was enunciated seeking support in the essay that Freyre published three years after Sérgio Buarque’s return. In the light of these considerations, Morse’s reflection points in the right direction, but it is insufficient, given that not only there is an influence but also it is extended (at least) until the fifth chapter of *Raízes* . André Furtado (2018) reconstructs similar circumstances going from the text to the context of *Raízes do Brasil* . All of this suggests one more challenge to the understanding of “writing of the self” by the author of *Tentativas de mitologia* (Attempts of Mithology) (see Carvalho, 2017 ).

3 Where possible, citations in English from *Raízes do Brasil* were sourced from Sérgio Buarque de Holanda, *Roots of Brazil* . Indiana: University of Notre Dame Press, 2012.
